# Sialendoscopy for treatment of major salivary glands diseases: a comprehensive analysis of published systematic reviews and meta-analyses

**DOI:** 10.1016/j.bjorl.2023.101293

**Published:** 2023-07-15

**Authors:** Lucas Kallas-Silva, Maria Fernanda Dias Azevedo, Fátima Cristina Mendes de Matos, Silvia Picado Petrarrolha, Rogério Aparecido Dedivitis, Marco Aurélio Vamondes Kulcsar, Leandro Luongo Matos

**Affiliations:** a Faculdade Israelita de Ciências da Saúde Albert Einstein, Serviço de Cirurgia de Cabeça e Pescoço, São Paulo, SP, Brazil; b Universidade de Pernambuco (UPE), Pernambuco, PE, Brazil; c Vice-presidente da Sociedade Brasileira de Cirurgia de Cabeça e Pescoço, Brazil; d Hospital das Clínicas da Faculdade de Medicina da Universidade de São Paulo (HCFMUSP), Departamento de Cirurgia (Cirurgia de Cabeça e Pescoço), São Paulo, SP, Brazil; e Ex-presidente da Sociedade Brasileira de Cirurgia de Cabeça e Pescoço, Brazil; f Hospital das Clínicas da Faculdade de Medicina da Universidade de São Paulo (HCFMUSP), Instituto do Câncer do Estado de São Paulo (Icesp), Cirurgia de Cabeça e Pescoço, São Paulo, SP, Brazil; g Presidente da Sociedade Brasileira de Cirurgia de Cabeça e Pescoço, Brazil; h Faculdade Israelita de Ciências da Saúde Albert Einstein, Clínica Cirúrgica, São Paulo, SP, Brazil; i Diretor Científico da Sociedade Brasileira de Cirurgia de Cabeça e Pescoço, Brazil

**Keywords:** Salivary gland, Sialadenitis, Salivary gland calculi, Sialendoscopy, Evidence-based practice

## Abstract

**Objectives:**

Sialendoscopy is a minimally invasive procedure used to diagnose and treat obstructive salivary gland diseases. Previous studies in the topic have shown mixed results. The present study aimed to evaluate the efficacy and safety of sialendoscopy through previous systematic reviews for different outcomes of several diseases. We also aimed to assess studies’ methodological quality and heterogeneity.

**Methods:**

We conducted a comprehensive systematic literature search of Pubmed, Embase, Lilacs and Cochrane Library. We included systematic reviews and meta-analyses that used sialendoscopy to treat both lithiasic and alithiasic salivary glands diseases. Data extraction included studies’ characteristics and results. We assessed studies’ methodological quality using the AMSTAR-2 (A MeaSurement Tool to Assess systematic Reviews 2) tool.

**Results:**

13 studies were included in the review, being 9 in adult populations and 4 in pediatric populations. Sialendoscopy proved to be effective at the treatment of different lithiasic and other obstructive diseases, but with important heterogeneity. The technique was also considered highly safe in most studies. However, studies had a critically low quality of evidence.

**Conclusions:**

Most studies demonstrated high efficacy and safety of sialendoscopy, but with critically low quality of evidence. We still lack randomized studies in this field, and future systematic reviews on the topic should follow current guidelines to improve conduction and reporting.

## Highlights

• Sialendoscopy was effective and safe in obstructive salivary glands diseases.
• Although it was an effective intervention, studies showed important heterogeneity.
• All reviews had critically low quality of evidence when using the AMSTAR-2 tool.
• We still lack comparative observational and interventional studies in sialendoscopy.
• Future reviews should follow guidelines to improve study conduction and reporting.

## Introduction

Salivary gland obstruction affects approximately 1% of the general population. Common symptoms include pain and edema that worsen when eating. Sialolithiasis is responsible for 60%–70%^1^ of all salivary gland obstructions. When the stone is large enough to obstruct the salivary duct, there is accumulation of saliva, with eventual chronic inflammatory response. Persistence of the obstruction is a risk factor for retrograde infections because of stagnation of saliva.

Sialendoscopy can be used to manage both lithiasic and alithiasic diseases of salivary glands, also known as Obstructive Salivary Gland Diseases (OSGDs), and for diagnosis, treatment, and assistance in surgery. Until recently, treatment of sialadenitis in symptomatic cases consisted of gland excision, with an inherent risk for adverse events.^2^ Asymptomatic or oligosymptomatic cases usually have conservative management, with satisfactory preservation of gland function, but with risk of salivary duct distension and persistence of symptoms due to saliva stagnation.

In the last few decades, minimally invasive techniques have been developed to treat symptomatic lithiasic and alithiasic salivary gland obstructions. Sialendoscopy uses small semi-rigid or semi-flexible endoscopes to access salivary ducts orally and visualize its’ lumen. It was introduced as an alternative to surgical removal of salivary glands, reducing morbidity related to the procedure and preserving the salivary glands. Many studies have shown sialendoscopy as a viable option for removal of stones in salivary ducts. It can also be used in children to treat juvenile recurrent parotitis, although smaller salivary ducts in children are a complicating factor. For instance, sialendoscopy has been used as the preferred method to treat both lithiasic and alithiasic OSGDs in many countries.

There are several systematic reviews and meta-analyses assessing sialendoscopy in different lithiasic and alithiasic OSGDs in adults, adolescents, and children. Most of them have shown high efficacy and safety of the procedure. However, most studies evaluate different diseases of the salivary glands, applied to varied populations. There is no single study evaluating different outcomes in all OSDGs. Moreover, there is also unknown what is the overall quality of evidence of these published reviews. In the same way, there is high clinical heterogeneity between the different published studies what can cause confusion in the interpretation of these results.

The present study evaluated the efficacy of sialendoscopy to treat different OSGDs, lithiasic or alithiasic, analyzing all published systematic reviews and meta-analyses in the field. We also assessed all studies` results and evaluated methodological quality.

## Methods

### Eligibility criteria and data extraction

We made a systematic search using the terms (“sialendoscopy” OR “sialoendoscopy”) in the Medline database in PubMed, Embase, Lilacs and Cochrane Library. Systematic reviews and meta-analyses of clinical trials or observational studies of any language and date up to April 2022 were eligible for inclusion. We included studies with both lithiasic and alithiasic OSGDs. After the search, assessment for eligibility and data extraction were made by one reviewer (L.L.M.). Data extraction included cited studies, search strategy, language, period of search, databases searched, PICO strategy (Population, Intervention, Control and Outcome),^3^ use of the Preferred Reporting Items for Systematic Reviews and Meta-Analyses (PRISMA) guideline,^4^ reporting of publication bias, primary outcomes, type of statistical analysis, heterogeneity, and reporting of methodological quality.

Study characteristics were described by their search strategy, language, period of search, databases searched, other search strategies, use of PRISMA, reporting of publication bias, number of included articles, population, intervention, outcome, reporting of quality of evidence, main results, and reporting of heterogeneity. These characteristics were summarized in Tables.

Methodological quality was assessed using the AMSTAR-2 tool.^5^ AMSTAR-2 is a critical appraisal tool for systematic reviews that include both randomized and non-randomized studies. It is used to assess quality of evidence taking into account critical domains in construction and reporting of systematic reviews and based on that, rates the confidence in the results of the review as low, medium or high. The tool was used by two independent investigators (L.K.S. and M.F.D.). Lack of consensus in any item was resolved by a third author (L.L.M.).

AMSTAR-2 tool defines several critical domains that should be accounted for when evaluating study quality. These are: prior establishment of review methods through protocol (Item 2), use of a comprehensive literature search strategy (Item 4), list of exclusions with justifications for exclusions (Item 7), assessment of risk of bias (Item 9), use of appropriate method in meta-analyses (Item 11), interpretation and discussion of the impact of risk of bias in the results (Item 13), and assessment of publication bias (Item 15).

Other items are considered non-critical by the authors but are also important to be assessed for. According to AMSTAR-2 authors, studies with one critical flaw are considered of low quality. Studies with more than one critical flaw are considered of critically low quality. In case there are no critical flaws, studies have moderate quality if they have more than one non-critical flaw and have high quality if they have one or no non-critical flaw.

We also summarized studies’ results separately for studies that included only lithiasic or alithiasic OSGDs, as well as studies that included both lithiasic and alithiasic OSGDs in their analyses. PRISMA reporting guideline was used in manuscript preparation.

## Results

### Study selection

**Figure 1:**
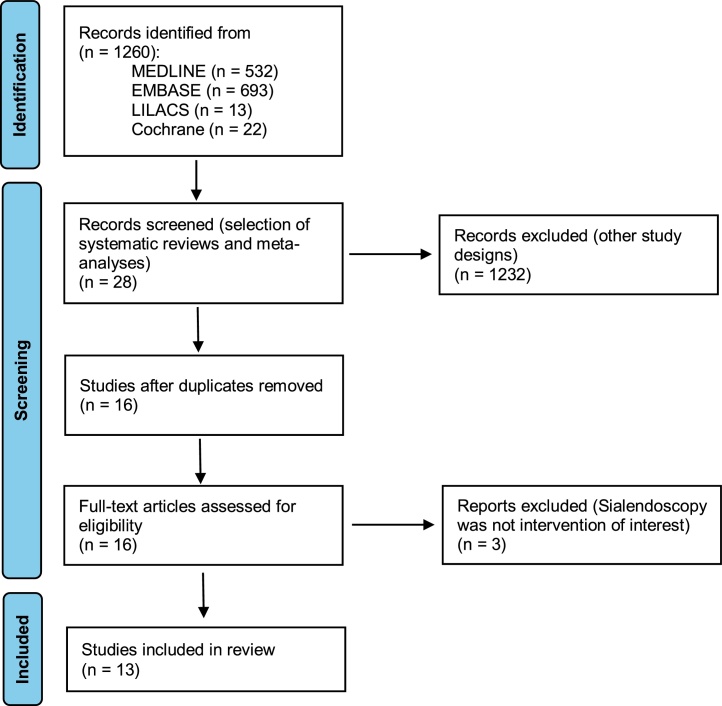
PRISMA flowchart of study inclusion.

With the presented search strategy, we identified a total of 1,260 studies. Of those, 28 were identified to be systematic reviews or meta-analyses. After exclusion of 12 duplicate studies, 16 studies were assessed by full text. Three articles were excluded because sialendoscopy was not the intervention of interest and 13 studies^1^, ^2^, ^6^, ^7^, ^8^, ^9^, ^10^, ^11^, ^12^, ^13^, ^14^, ^15^, ^16^ were included in the final analysis. A flowchart of the inclusion of studies is presented in Fig. 1.

### Study characteristics

Most of the assessed systematic reviews and meta-analyses included only observational studies,^1^, ^2^, ^6^, ^7^, ^8^, ^9^, ^10^, ^11^, ^13^, ^15^, ^16^ with most of them being retrospective studies. All of them reported their search strategy and all but one,^9^ reported search period. Most of them searched through a variety of databases, except for two,^12^, ^13^, ^14^, ^15^ which included only pubmed. Seven studies described the use of PRISMA guideline for reporting results.^6^, ^8^, ^10^, ^12^, ^13^, ^14^, ^16^

The population evaluated in different studies significantly varied. Of the nine studies in adult populations, four studies included only lithiasic OSGDs,^1^, ^7^, ^13^, ^15^ one study included OSGD with underlying Sjogren syndrome,^14^ one study included radioiodine induced sialadenitis^10^ and three studies included both lithiasic and alithiasic OSGDs.^6^, ^9^, ^16^ Of the 4 studies in children and adolescents, two included only lithiasic OSGDs^9^, ^11^ and two included juvenile recurrent parotitis.^8^, ^12^ All of them included use of sialendoscopy as an intervention, although some included associated interventions, such as corticosteroid use,^14^, ^16^ saline solution,^14^ and other medication treatments.^12^ In two studies, the intervention was surgery combined to sialendoscopy assistance.^1^, ^7^ The studies assessed different outcomes: sialendoscopy effectiveness,^1^, ^2^, ^6^, ^9^^,^^11^, ^16^ symptoms resolution,^7^, ^8^, ^10^, ^13^, ^14^, ^15^, ^16^ safety and adverse events1,^6^, ^11^ recurrence of the disease or symptoms,^12^ and salivary gland preservation.^13^

Study PICO strategy is shown in Table 1. We did not report control analyses because most primary studies had no comparator. As a result, when studies evaluated efficacy, they reported success rate of sialendoscopy, recurrence rate, or symptom resolution. When evaluating safety, studies reported minor and major complications. Study characteristics are shown in Table 2. We also evaluated what primary studies were cited in each systematic review and meta-analysis using a citation matrix (Table S1; Supplementary Material). As noted, there was a large variability in the primary studies included in the reviews, although pediatric studies were more likely to include the same primary studies in their reviews.

**Table 1:** PICO strategy used in included studies.

Study	Population	Intervention	Outcome
Lithiasic obstructive sialadenitis			
*Studies with adult population			
Jadu, 2014	Adults with obstructive sialadenitis (lithiasic)	Sialendoscopy-assisted stone removal surgery	Symptom resolution and residual sialolithiasis
Roland, 2017	Adults with obstructive sialadenitis (lithiasic)	Sialendoscopy-assisted parotid stone removal surgery	Efficacy and safety of sialendoscopy-assisted parotid stone surgical removal
Chiesa-Estomba, 2020	Adults with obstructive sialadenitis (lithiasic)	Sialendoscopy associated with laser-assisted lithotripsy	Symptom resolution and glandular preservation rate
Galdermans, 2020	Patients with parotid sialolithiasis	Sialendoscopy or sialolithotripsy alone and a combination of both techniques	Partial or complete symptom improvement
*Studies with pediatric population			
Silva, 2016	Children and teenagers with obstructive sialadenitis (lithiasic and alithiasic)	Sialendoscopy	Effectiveness of sialendoscopy
Schwarz, 2017	Children and teenagers with obstructive sialadenitis (lithiasic and alithiasic)	Sialendoscopy	Efficacy and side effects
Lithiasic or alithiasic obstructive sialadenitis			
*Studies with adult population			
Strychowsky, 2012	Adults with obstructive sialadenitis (lithiasic and alithiasic)	Sialendoscopy	Efficacy and safety of sialendoscopy
Atienza, 2015	Adults with obstructive sialadenitis (lithiasic and alithiasic)	Sialendoscopy	Obstruction resolution (sialendoscopy alone or combinated)
Donaldson, 2021	Adults with obstructive sialadenitis (lithiasic and alithiasic)	Sialendoscopy associated with oral corticosteroids	Partial or complete symptom improvement or lithiasis resolution
Alithiasic obstructive sialadenitis			
*Studies with adult population			
Cung, 2017	Adults with radioactive iodine-induced sialoadenitis refractory to medical treatment	Sialendoscopy	Clinical improvement (symptom reduction)
Coca, 2020	Symptomatic sialoadenitis due to Sjogren's syndrome	Corticoid or saline solution associated sialendoscopy	Partial or complete symptom improvement
*Studies with pediatric population			
Ramakrishna, 2014	Children and teenagers with juvenile recurrent parotitis	Sialendoscopy	Symptom resolution
Garavello, 2018	Children and teenagers with juvenile recurrent parotitis	Sialendoscopy and other drug treatments	Sialadenitis recurrence rate

**Table 2:** Methodological characteristics of included studies.

Study	Language	Period	Databases	Other search methods	Included articles	Study designs reported	PRISMA use
Lithiasic obstructive sialadenitis							
*Studies with adult populations							
Jadu, 2014	English	2004 to 2013	MEDLINE, EMBASE, and Cochrane Library	References of included studies	11	Not reported	No
Roland, 2017	English	Up to March 2015	PubMed, Embase, Cumulative Index to Nursing, Allied Health Literature and the Cochrane Database of Systematic Reviews	Search for non-published data	10	Primarily retrospective (not specified how many prospective or retrospective)	No
Chiesa-Estomba, 2020	English, German, French and Spanish	Up to 2020	PubMed, Google Scholar, and Scopus	?	16	11 retrospective and 5 prospective (non-randomized)	Yes
Galdermans, 2020		January 2007 to January 2017	Pubmed	?	13	10 case series (7 retrospective and 3 prospective), 1 case-control, 1 observational and 1 evaluation study	?
*Studies with pediatric populations							
Silva, 2016	English, Italian, Portuguese and Spanish	?	Pubmed, Scielo, and Cochrane	?	7	7 case series reports	?
Schwarz, 2017	English, Italian, French and Spanish	January 1990 to January 2017	Africa-Wide Information, Biosis (Previews 1969–2016), Cochrane, Embase (from 1947), LILACs, Medline (from 1946), PubMed, and Web of science	References of included studies	17	17 case series (15 retrospective and 2 prospective)	No
Lithiasic or alithiasic obstructive sialadenitis							
*Studies with adult populations							
Strychowsky, 2012	English	Up to October 2010	MEDLINE, EMBASE, and Cochrane Library	References of included studies	29	Not reported	Yes
Atienza, 2015	English, French, Italian, Portuguese and Spanish	Up to April 2014	MEDLINE, EMBASE, ISI Web of Knowledge, The Cochrane Library, and the NHS Centre for Reviews and Dissemination	References of included studies	49	Not reported	?
Donaldson, 2021	English	Up to September 2020	PUBMED, EMBASE, PROQUEST, and Cochrane Library	?	9	8 case series and 1 prospective comparative study	Yes
Alithiasic obstructive sialadenitis							
*Studies with adult populations							
Cung, 2017	English	Up to April 2017	MEDLINE, EMBASE, and Cochrane Library	?	8	6 retrospective and 2 prospective studies	Yes
Coca, 2020	English	Up to August 2020	PubMed, Cochrane, and Scopus	?	6	2 RCTs and 4 case series	Yes
*Studies with pediatric populations							
Ramakrishna, 2014	English	Up to November 2013	MEDLINE, EMBASE, Cochrane Library, and Google Scholar	References of included studies	7	4 cohort studies and 3 studies with no comparator	Yes
Garavello, 2018	English	January 1990 to April 2018	Pubmed	References of included studies	19	1 RCT, 2 studies with comparator, 20 retrospective case series and 1 case report	Yes

### Overview of study results

In general, the sialendoscopy method was referred to as effective and well tolerated. Individual study results are presented in Table 3. In studies evaluating exclusively adults with only lithiasic OSGDs, success rate in stone removal was highest in Roland (2017),^1^ and all other reviews identified high success rate, although Jadu (2014)^7^ included studies with moderate heterogeneity of success rates. Symptom improvement and resolution was high, with a low rate of complications. In children and adolescents, although studies had a very low quality of evidence, sialendoscopy was effective with high improvement of symptoms.

**Table 3:** Main results of included studies and heterogeneity.

Article	Main results	Heterogeneity	Assessment of quality of evidence
Lithiasic obstructive sialadenitis			
*Studies with adult populations			
Jadu, 2014	Success rate range: 69%–100%	Moderate	Not evaluated
	Very few complications		
	Grouped success rate: 92.8% (95% CI 87–96)		
Roland, 2017	Stone removal rate: 99% (95% CI 97–100)	Low (moderate for complications)	Moderate (Gu et al. J Clin Epidemiol 2016; 69:199–207.e192)
	Symptom improvement: 97% (95% CI 93–99)		
	Gland preservation: 100% (95% CI 99–100)		
	Complications: 6% (95% CI 1–15)		
Chiesa-Estomba, 2020	(1) Resolution of obstruction: 87.3% (95% CI 71–100)	No meta-analyses	Low (National Institute for Health and Clinical Excellence)
	(2) Gland preservation: 97%		
	(3) Adverse events <3%		
Galdermans, 2020	(1) Mean success rate 88.7% (range 71.4%–100%)	No meta-analyses	Not evaluated
	(2) Low rate of complications and no major complications		
*Studies with pediatric populations			
Silva, 2016	Efficacy range: 83%–93%	No meta-analyses	Not evaluated
Schwarz, 2017	Reccurency rate (symptom or stone): 14.5%, most of them in JRP	No meta-analyses	Not evaluated
Lithiasic or alithiasic obstructive sialadenitis			
*Studies with adult populations			
Strychowsky, 2012	(1) Success of sialendoscopy alone: 86% (95% CI 83–89)	High (1)/Low (2)	Not evaluated
	(2) Sucess of sialendoscopy with combined access: 93% (95% CI 89–96)		
	(3) Success range in radioiodine-induced sialadenitis: 50%–100%		
	(4) Need of gland ressection range: 0%–11%		
	Few major complications		
Atienza, 2015	(1) Resolution of obstruction in sialendoscopy alone: 76% (95% CI 71%−82%)	High	Low (SIGN ‒ Scottish Intercollegiate Guidelines Network)
	Resolution of obstruction in open surgery with sialendoscopy: 91% (95% CI 88–94)		
	(2) Gland preservation rate range in sialendoscopy alone: 75.5%–100%		
	Gland preservation rate range in open surgery with sialendoscopy: 66.7%–100%		
Donaldson, 2021	Success rate: 89%	No meta-analyses	Low (qualitative; reference not specified)
Alithiasic obstructive sialadenitis			
*Studies with adult populations			
Cung, 2017	Clinical improvement range: 75%–100%	No meta-analyses	Moderate (GRADE)
Coca, 2020	(1) No estimation symptom resolution alone	High (1)/Low (2)	High/Moderate (National Institute for Health and Clinical Excellence)
	(2) Symptom resolution or symptom improvement: 95% (95% CI 90–99)		
*Studies with pediatric populations			
Ramakrishna, 2014	(1) Grouped success rate (no recurrence of sialadenitis): 73% (95% CI 64–82)	Low	Not evaluated
	Success rate by gland (no recurrence of sialadenitis): 81% (95% CI 75–87)		
	Grouped rate of patients with no necessity of other sialendoscopy: 87% (95% CI 81–93)		
Garavello, 2018	(1) Recurrece rate: 25.8% (95% CI 21.5–30.8)	Not evaluated	Low (qualitative; reference not specified)
	(2) Success rate: 74.2% (95% CI 69.2–78.5)		

In studies including both lithiasic and alithiasic OSGDs in adults, we identified a large heterogeneity of outcomes, which makes it difficult to interpret their individual results. Although success rates of sialendoscopy alone was lower in Strychowsky (2012)^6^ and Atienza (2015),^2^ there was a high success rate when sialendoscopy was used combined with open surgery. Donaldson (2021)^16^ had a high success rate. All three studies had few adverse events.

In studies analyzing specifically alithiasic OSGDs in adult populations, Cung (2017)^10^ identified a high clinical improvement in patients with radioiodine-induced sialadenitis, with moderate quality of evidence and heterogeneity (although meta-analysis was not conducted). Coca (2020)^14^ identified a high clinical improvement in patients with underlying Sjogren’s syndrome. In children and adolescents with juvenile recurrent parotitis, success rate (as defined by absence of recurrence) was moderate in both studies.

### Assessment of methodological quality

Assessment of methodological quality using the AMSTAR-25,^17^, ^18^ tool is shown in Table 4. Since we included studies regarding different populations, we will present the results according to the research question. Although not evaluated through the AMSTAR-2 tool, it is important to note that most systematic reviews and meta-analyses included low quality primary studies, most of them being observational retrospective studies. Few studies included Randomized Controlled Trials (RCTs).

**Table 4:** Assessment of methodological quality of studies using the AMSTAR-2 tool.

	Lithiasic obstructive sialadenitis						Lithiasic and alithiasic obstructive sialadenitis			Alithiasic obstructive sialadenitis				Number of Yes or Partially Yes
	Adults				Children and adolescents		Adults			Adults		Children and adolescents		
	Jadu, 2014	Roland, 2017	Chiesa-Estomba, 2020	Galdermans, 2020	Silva, 2016	Schwarz, 2017	Strychowsky, 2012	Atienza, 2015	Donaldson, 2021	Cung, 2017	Coca, 2020	Ramakrishna, 2014	Garavello, 2018	
1) Research questions and criteria (PICO)	Yes	Yes	Yes	Yes	Yes	Yes	Yes	Yes	Yes	Yes	Yes	Yes	Yes	13/13
2) Prior establishment of review methods	No	No	No	No	Partial yes	No	No	No	No	No	No	No	No	1/13
3) Study design selection explanation	No	Yes	Yes	Yes	Yes (in protocol)	Yes	Yes	No	Yes	Yes	Yes	Yes	No	10/13
4) Comprehensive literature search	Partial yes	Partial yes	Partial yes	No	Partial yes	Partial yes	Partial yes	Partial yes	Partial yes	Partial yes	Partial yes	Partial yes	No	11/13
5) Study selection in duplicate	Yes	Yes	Yes	No	Yes	Yes	Yes	Yes	Yes	No	Yes	Yes	Yes	11/13
6) Data extraction in duplicate	Yes	Yes	Yes	No	Yes	Yes	Yes	Yes	Yes	No	Yes	Yes	Yes	11/13
7) Justification of exclusions	Partial yes	Yes	Yes	Yes	No	Yes	Yes	Partial yes	Partial yes	No	Yes	Yes	Partial yes	11/13
8) Adequate detail describing included studies	No	Yes	Yes	Yes	No	Yes	No	No	Yes	Yes	Yes	Yes	Partial yes	9/13
9) Risk of bias assessment in RCTs and NRCTs	No	Partial yes	Partial yes	No	No	No	No	No	No	Partial yes	Yes	No	No	4/13
10) Sources of funding in include studies	No	No	No	No	No	No	No	No	No	No	No	No	No	0/13
11) Use of appropriate statistical methods in RCTs and NRCTs	Yes	Yes	No meta-analyses	No meta-analyses	No meta-analyses	No meta-analyses	Yes	Yes	No meta-analyses	No meta-analyses	Yes	Yes	No	6/13
12) Risk of bias impact assessment	No	Yes	No meta-analyses	No meta-analyses	No meta-analyses	No meta-analyses	No	No	No meta-analyses	No meta-analyses	Yes	No	No	2/13
13) Risk of bias when interpreting or discussing results	No	Yes	Yes	No	No	No	No	No	Yes	Yes	Yes	No	No	5/13
14) Explanation and discussion of heterogeneity	No	Yes	Yes	No	No	No	Yes	Yes	No	No	Yes	No	No	5/13
15) Adequate investigation of publication bias	Yes	No	No meta-analyses	No meta-analyses	No meta-analyses	No meta-analyses	No	No	No meta-analyses	No meta-analyses	No	Yes	No	2/13
16) Sources of conflict of interest	Yes	Yes	Yes	Yes	Yes (in protocol)	Yes	Yes	Yes	Yes	Yes	Yes	Yes	Yes	13/13
Yes in all domains	6/16	11/16	9/16	5/16	5/16	7/16	8/16	6/16	7/16	5/16	12/16	9/16	4/16	
Yes in critical domains	2/7	3/7	2/7	1/7	0/7	1/7	2/7	1/7	1/7	1/7	4/7	3/7	0/7	
Rating	Critically low	Critically low	Critically low	Critically low	Critically low	Critically low	Critically low	Critically low	Critically low	Critically low	Critically low	Critically low	Critically low	

Overall compliance was 46% (range 25%–75%), and compliance in critical domains was 23% (range 0%–57%). All reviews had critically low quality of evidence. Critical domains with the lowest compliance were previous protocol registration (Item 2) and assessment of publication bias (Item 15). The only domain with no compliance was reporting of funding in individual studies, which is a non-critical domain.

Of four studies in adult populations that included only lithiasic OSGDs, Roland (2017)^1^ and Chiesa-Estomba (2020)^13^ had higher methodological quality in adult populations. When regarding lithiasic OSGDs in pediatric populations, Schwarz (2017)^11^ had a slightly higher methodological quality than Silva (2016).^9^ In studies that included both lithiasic and alithiasic OSGDs, the three included studies had very similar methodological quality in adults. In studies with only alithiasic OSGDs in adults, Coca (2020)^14^ had a higher methodological quality, although it included only OSGDs with underlying Sjogren syndrome. Ramakrishna (2014)^8^ had a higher methodological quality in the investigation of juvenile recurrent parotitis.

## Discussion

In the present study, we assessed the efficacy and safety of sialendoscopy to treat several OSGDs in different populations and also in different clinical scenarios. Our analysis included all systematic reviews and meta-analyses in the topic. Studies’ overall results and methodological quality were assessed. According to the available evidence, sialendoscopy has shown to be an effective and safe technique to treat OSGDs. However, we found that all systematic reviews published in the topic have critically low quality of evidence, when assessed by the AMSTAR-2 checklist. The present study provides evidence-based guidance for clinical practice, considering different populations, several diseases, and outcomes.

There are several evidence-based studies published in the literature considering sialendoscopy in the treatment of OSGDs. These studies include systematic reviews with or without meta-analysis with different populations and different outcomes. These studies, sometimes, evaluate the same outcome, but with different inclusion criteria, making the comparison between them sometimes impossible and also demonstrating divergent results. Moreover, the lack of an objective analysis in terms of the quality of the generated evidence, makes it impossible to safely employ these results into clinical practice.

Most analyzed publications included primarily retrospective studies, resulting in low to moderate quality of evidence. There are important biases to consider when analyzing retrospective studies, such as recall bias in subjective outcomes and lack of randomization and close follow-up of patients. Short follow-up was also an important limitation of the primary studies, hindering our capability of identifying long-term symptoms. Study outcomes were assessed and reported differently, adding more bias and heterogeneity. Symptom improvement may be the most important outcome since it is a clinical outcome, but lack of validated standardized methods for evaluating symptoms makes it harder to define what is a relevant improvement. Thus, bias was largely present in our evaluation, which importantly limits reliability of results in studies of sialendoscopy.

Heterogeneity played an important role in our analyses. Generally, included reviews had moderate heterogeneity in their data. This could be due to small sample sizes, variability in technique and surgical equipment and inconsistent reporting. Most meta-analyses included several case reports and case series. These study designs should not be used in meta-analyses, due to large variability of methodology. This also adds heterogeneity to the studies. There was also large heterogeneity in our review, since most studies included different populations, interventions, and outcomes. Even when included reviews evaluated the same research question, there was important heterogeneity in their results, which we attribute to variable methodological quality. The presence of many sources of heterogeneity limits the interpretability and generalizability of all included reviews.

A point should be made regarding the separation of the analysis between lithiasic and alithiasic OSGDs. As noted, 3 studies included both lithiasic and alithiasic OSGDs in their analyses. We opted to include these studies to guarantee that all available data in OSGDs was covered. Moreover, since there was large overlap of primary studies included in the reviews, we cannot properly evaluate the accuracy of sialendoscopy through a simple division of lithiasic and alithiasic OSGDs. However, this does not impact the quality of evidence of presented in each population, since the critically low methodological quality was present in all studies.

Methodological quality of reviews of studies in sialendoscopy was also an important limitation. All included studies had a critically low methodological quality, according to our assessment using the AMSTAR-2 tool, making this point one of the largest weaknesses in the outcome assessment of sialendoscopy technique. Studies lacked especially critical domains, leading to poor ratings. According to AMSTAR-2, studies should be classified with critically low quality when they have more than one critical flaw. The great majority of studies did not include Items 2 (prior establishment of methods) and 15 (assessment for publication bias), considered to be critical, which already led to most of them having critically low quality. We also noted that PRISMA guideline use played an important role when assessing methodological quality. Studies that used it to report their results had generally higher quality of evidence.

Additionally, we would like to make some comments regarding our assessment of methodological quality. In Item 2, regarding prior establishment of review methods, we considered as a “no” when studies reported use of a protocol, but the protocol could not be found in supplementary material or in online platforms for protocol registration. Following AMSTAR-2 recommendations, studies that verified only one database received a “no” in Item 4, regarding search strategy. In Item 7, when authors did not report reading articles in full text, the review received a “no”. This is a point of caution that should be a warning for all authors that intend to conduct a systematic review, with or without a meta-analysis. The better the authors describe the methodology applied at the study, the better the quality score. We also strongly recommend that authors register their projects into specific platforms, assuring a high-quality publication.

Finally, it is important to note that some studies performed meta-analyses, whereas others do not. Naturally, studies with no meta-analyses have lower ratings since they do not score in some items. Even though this is a limitation, it is important to note that, even in other items that do not include only meta-analyses, studies that made meta-analyses also had less flaws.

More primary and secondary studies should be performed using sialendoscopy, with more rigid methodologies and less predictable bias, to better establish the method as gold-standard for OSGDs treatment.

## Conclusion

In this analysis of systematic reviews and meta-analyses, we found sialendoscopy to be efficacious and safe. However, the included studies showed critically low quality of evidence. We still lack randomized studies in this field, and future systematic reviews on the topic should follow current guidelines to improve conduction and reporting.

## Disclosures

The preliminary results of this work were presented at the 2022 American Academy of Otolaryngology - Head and Neck Society Foundation Annual Meeting as a poster.

## Financial disclosure

This research did not receive any specific funding from funding agencies in the public, commercial, or not-for-profit sectors.

## Conflicts of interest

The authors declare no conflicts of interest.
