# Supplementary material for: Sialendoscopy for treatment of major salivary glands diseases: a comprehensive analysis of published systematic reviews and meta-analyses
Source: Braz J Otorhinolaryngol. 2023 Jul 15;89(5):101293. doi: 10.1016/j.bjorl.2023.101293 (PMC10382863; doi:10.1016/j.bjorl.2023.101293)
Supplement: Supplementary file 1 [file mmc1.docx]

**BJORL-D-23-00089_** Supplementary Material

Supplementary Material Table 1 Citation matrix of primary studies included in previous systematic reviews.

|  | **Lithiasic obstructive sialadenitis** | | | | | | **Lithiasic or alithiasic obstructive sialadenitis** | | | **Alithiasic obstructive sialadenitis** | | | |
| --- | --- | --- | --- | --- | --- | --- | --- | --- | --- | --- | --- | --- | --- |
|  | **Adults** | | | | **Children and adolescents** | | **Adults** | | | **Adults** | | **Children and Adolescents** | |
| **Study** | **Jadu, 2014 [1]** | **Roland, 2017 [2]** | **Chiesa-Es tomba, 2020 [3]** | **Galderm ans, 2020 [4]** | **Silva, 2016 [5]** | **Schwarz, 2017 [6]** | **Strychowsky, 2012 [7]** | **Atienza, 2015 [8]** | **Donaldso n, 2021 [9]** | **Cung, 2017 [10]** | **Coca, 2020 [11]** | **Ramakrishna, 2014 [12]** | **Garavello, 2018 [13]** |
| Gundlach, 1990 [14] |  |  | X |  |  |  |  |  |  |  |  |  |  |
| Konigsberger, 1993 [15] |  |  |  |  |  |  | X | X |  |  |  |  |  |
| Arzoz, 1996 [16] |  |  |  |  |  |  | X | X |  |  |  |  |  |
| Ito, 1996 [17] |  |  | X |  |  |  |  |  |  |  |  |  |  |
| Nahlieli, 2000 [18] |  |  |  |  |  | X |  |  |  |  |  |  |  |
| Marchal, 2001 [19] |  |  |  |  |  |  | X | X | X |  |  |  |  |
| Marchal, 2002 [20] |  |  | X |  |  |  | X | X | X |  |  |  |  |
| Chu, 2003 [21] |  |  | X |  |  |  | X | X |  |  |  |  |  |
| Katz, 2004 [22] |  |  | X |  |  |  |  |  |  |  |  |  |  |
| Nahlieli, 2004 [23] |  |  |  |  |  | X |  |  |  |  |  |  | X |
| Zenk, 2004 [24] |  |  |  |  |  |  | X | X |  |  |  |  |  |
| Ziegler, 2004 [25] | X |  |  |  |  |  | X | X |  |  |  |  |  |
| Koch, 2005 [26] |  |  |  |  |  |  | X | X |  |  |  |  |  |
| McGurk, 2006 [27] | X |  |  |  |  |  | X | X |  | X |  |  |  |
| Raif, 2006 [28] |  |  | X |  |  |  | X | X |  |  |  |  |  |
| Faure, 2007 [29] |  |  |  |  | X | X |  |  |  |  |  |  |  |
| Kim, 2007 [30] |  |  |  |  |  |  | X |  |  | X |  |  |  |
| Marchal, 2007 [31] | X | X |  |  |  |  |  |  |  |  |  |  |  |
| Nahlieli, 2007 [32] | X |  |  |  |  |  | X | X |  |  |  |  |  |
| Koch, 2008 [33] |  |  |  |  |  |  | X | X |  |  |  |  |  |
| Modayil, 2008 [34] |  |  |  |  |  |  |  |  | X |  |  |  |  |
| Papadaki, 2008 [35] |  |  |  |  |  |  | X | X |  |  |  |  |  |
| Quenin, 2008 [36] |  |  |  |  |  | X |  |  |  |  |  | X | X |
| Schmitz, 2008 [37] |  |  |  | X |  |  |  |  |  |  |  |  |  |
| Walvekar, 2008 [38] |  |  |  |  |  |  | X | X |  |  |  |  |  |
| Yu, 2008 [39] |  |  |  |  |  |  | X | X |  |  |  |  |  |
| Yu, 2008a [40] |  |  |  |  |  |  |  | X |  |  |  |  |  |
| Bomeli, 2009 [41] |  |  |  |  |  |  | X |  |  | X |  |  |  |
| Iro, 2009 [42] |  |  |  | X |  |  |  |  |  |  |  |  |  |
| Liu, 2009 [43] | X |  |  |  |  |  | X | X |  |  |  |  |  |
| Nahlieli, 2009 [44] |  |  |  |  |  |  | X | X |  |  |  |  |  |
| Shacham, 2009 [45] |  |  |  |  | X | X |  |  |  |  |  |  |  |
| Walvekar, 2009 [46] | X |  |  |  |  |  | X | X |  |  |  |  |  |
| Ardekian, 2010 [47] |  |  |  |  |  |  |  | X |  |  |  |  |  |
| Escudier, 2010 [48] |  |  |  | X |  |  |  |  |  |  |  |  |  |
| Jabbour, 2010 [49] |  |  |  |  | X | X |  |  |  |  |  | X | X |
| Karavidas, 2010 [50] | X | X |  |  |  |  | X | X |  |  |  |  |  |
| Koch, 2010 [51] | X |  |  |  |  |  | X | X |  |  |  |  |  |
| Martins-Carvalho, 2010 [52] |  |  |  |  | X | X |  |  |  |  |  |  |  |
| Nahlieli, 2010 [53] |  |  |  |  |  |  | X | X |  |  |  |  |  |
| Serbetci, 2010 [54] |  |  |  | X |  |  | X | X |  |  |  |  |  |
| Su, 2010 [55] | X |  |  |  |  |  | X | X |  |  |  |  |  |
| Wallace, 2010 [56] | X |  |  |  |  |  | X | X |  |  |  |  |  |
| Yu, 2010 [57] |  |  |  |  |  |  | X | X |  |  |  |  |  |
| Bowen, 2011 [58] |  |  |  |  |  |  |  | X |  |  |  |  |  |
| Capaccio, 2011 [59] |  |  |  |  |  |  |  | X |  |  |  |  | X |
| Danquart, 2011 [60] |  |  |  |  |  |  |  | X |  |  |  |  |  |
| Gary, 2011 [61] |  |  |  |  |  | X |  |  |  |  |  | X | X |
| Gillespie, 2011 [62] |  |  |  |  |  |  |  | X |  |  |  |  |  |
| Konstantinidis, 2011 [63] |  |  |  |  |  | X |  |  |  |  |  | X | X |
| Luers, 2011 [64] |  |  |  |  |  |  |  | X |  |  |  |  |  |
| Maresh, 2011 [65] |  |  |  |  |  |  |  | X |  |  |  |  |  |
| Shacham, 2011 [66] |  |  |  |  |  |  |  |  |  |  | X | X | X |
| Capaccio, 2012 [67] |  |  |  |  | X | X |  |  |  |  |  | X |  |
| Durbec, 2012 [68] |  |  | X | X |  |  |  | X |  |  |  |  |  |
| Hackett, 2012 [69] |  |  |  |  | X | X |  |  |  |  |  | X | X |
| Koch, 2012 [70] |  |  |  |  |  |  |  | X |  |  |  |  |  |
| Koch, 2012a [71] |  |  |  |  |  |  |  | X |  |  |  |  |  |
| Kopec, 2012 [72] |  |  |  | X |  |  |  |  |  |  |  |  |  |
| Prendes, 2012 [73] |  |  |  |  |  |  |  |  |  | X |  |  |  |
| Rasmussen, 2012 [74] |  |  |  |  |  |  |  | X |  |  |  |  |  |
| Zenk, 2012 [75] |  |  |  | X |  |  |  | X |  |  |  |  |  |
| Carroll, 2013 [76] |  | X |  |  |  |  |  |  |  |  |  |  |  |
| Koch, 2013 [77] |  | X |  |  |  |  |  |  |  |  |  |  |  |
| Kopec, 2013 [78] |  |  |  |  |  |  |  | X |  |  |  |  |  |
| Kopec, 2013a [79] | X | X |  |  |  |  |  | X |  |  |  |  |  |
| Kopec, 2013b [80] |  |  |  |  |  |  |  | X |  |  |  |  |  |
| Kroll, 2013 [81] |  |  |  |  |  |  |  | X |  |  |  |  |  |
| Martelucci, 2013 [82] |  |  | X |  |  |  |  |  |  |  |  |  |  |
| Meyer, 2013 [83] |  |  |  |  |  |  |  |  | X |  |  |  |  |
| Schneider, 2013 [84] |  |  |  |  |  |  |  |  |  |  |  |  | X |
| Vashishta, 2013 [85] |  |  |  |  |  |  |  | X |  |  |  |  |  |
| Ardekian, 2014 [86] |  |  |  |  | X | X |  | X |  |  |  |  | X |
| Ardekian, 2014a [87] |  |  |  |  |  |  |  | X | X |  |  |  |  |
| Capaccio, 2014 [88] |  | X |  |  |  |  |  |  |  |  |  |  |  |
| De Luca, 2014 [89] |  |  |  |  |  |  |  |  |  | X |  |  |  |
| Desmots, 2014 [90] |  |  |  | X |  |  |  |  |  |  |  |  |  |
| Ianovski, 2014 [91] |  |  |  |  |  |  |  | X |  |  |  |  |  |
| Klein, 2014 [92] |  | X |  |  |  |  |  | X |  |  |  |  |  |
| Konstantinidis, 2014 [93] |  | X |  |  |  |  |  |  |  |  |  |  |  |
| Mikolajczak, 2014(94) |  |  |  |  |  | X |  |  |  |  |  |  | X |
| Numminen, 2014 [95] |  | X |  |  |  |  |  |  | X |  |  |  |  |
| Phillips, 2014 [96] |  |  | X | X |  |  |  | X |  |  |  |  |  |
| Semensohn, 2014 [97] |  |  |  |  |  | X |  |  |  |  |  |  | X |
| Sionis, 2014 [98] |  |  | X | X |  |  |  | X |  |  |  |  |  |
| Bhayani, 2015 [99] |  |  |  |  |  |  |  |  |  | X |  |  |  |
| De Luca, 2015 [100] |  |  |  |  |  |  |  |  |  |  | X |  |  |
| Mikolajczak, 2015 [101] |  | X |  |  |  |  |  |  |  |  |  |  |  |
| Nahlieli, 2015 [102] |  |  |  | X |  |  |  |  | X |  |  |  |  |
| Papadopoulou-Alataki, 2015 [103] |  |  |  |  |  | X |  |  |  |  |  |  | X |
| Rosbe, 2015 [104] |  |  |  |  |  |  |  |  |  |  |  |  | X |
| Su, 2015 [105] |  |  | X |  |  |  |  |  |  |  |  |  |  |
| Wu, 2015 [106] |  |  |  |  |  |  |  |  |  | X |  |  |  |
| Cordesmeyer, 2016 [107] |  |  |  | X |  |  |  |  |  |  |  |  |  |
| Honnet, 2016 [108] |  |  |  |  |  | X |  |  |  |  |  |  | X |
| Jager, 2016 [109] |  |  |  |  |  |  |  |  |  |  | X |  |  |
| Kim, 2016 [110] |  |  |  |  |  |  |  |  |  | X |  |  |  |
| Koch, 2016 [111] |  |  |  | X |  |  |  |  |  |  |  |  |  |
| Su, 2016 [112] |  |  | X |  |  | X |  |  |  |  |  |  | X |
| Achim, 2017 [113] |  |  | X |  |  |  |  |  |  |  |  |  |  |
| Capaccio, 2017 [114] |  |  |  |  |  |  |  |  |  |  |  |  | X |
| Carta, 2017 [115] |  |  | X |  |  |  |  |  |  |  |  |  |  |
| Guo, 2017 [116] |  |  |  |  |  |  |  |  |  |  | X |  |  |
| Singh, 2017 [117] |  |  |  |  |  |  |  |  |  |  |  |  | X |
| Bawazeer, 2018 [118] |  |  |  |  |  |  |  |  | X |  |  |  |  |
| Berlucchi, 2018 [119] |  |  |  |  |  |  |  |  |  |  |  |  | X |
| Capaccio, 2018 [120] |  |  |  |  |  |  |  |  |  |  | X |  |  |
| Karagozoglu, 2018 [121] |  |  |  |  |  |  |  |  |  |  | X |  |  |
| Guenzel, 2019 [122] |  |  | X |  |  |  |  |  |  |  |  |  |  |
| Ozçlik, 2019 [123] |  |  | X |  |  |  |  |  |  |  |  |  |  |
| De Paiva-Leite, 2021 [124] |  |  |  |  |  |  |  |  | X |  |  |  |  |

References

1. Jadu FM, Jan AM. A meta-analysis of the efficacy and safety of managing parotid and submandibular sialoliths using sialendoscopy assisted surgery. Saudi Med J. 2014;35(10). www.smj.org.sa.

2. Roland LT, Skillington SA, Ogden MA. Sialendoscopy-assisted transfacial removal of parotid sialoliths: A systematic review and meta-analysis. Laryngoscope. 2017;127(11):2510-2516. doi:10.1002/lary.26610.

3. Chiesa-Estomba CM, Saga-Gutierrez C, Calvo-Henriquez C, et al. Laser-Assisted Lithotripsy With Sialendoscopy: Systematic Review of YO-IFOS Head and Neck Study Group. Ear, Nose and Throat Journal. 2021;100(1_suppl):42S-50S. doi:10.1177/0145561320926281.

4. Galdermans M, Gemels B. Success rate and complications of sialendoscopy and sialolithotripsy in patients with parotid sialolithiasis: a systematic review. Oral and Maxillofacial Surgery. 2020;24(2):145-150. doi:10.1007/s10006-020-00834-x.

5. Silva L, Babicsak G, Dolci RL. Salivary gland endoscopy in children: A systematic review. Revista da Associacao Medica Brasileira. 2016;62(8):795-799. doi:10.1590/1806-9282.62.08.795.

6. Schwarz Y, Bezdjian A, Daniel SJ. Sialendoscopy in treating pediatric salivary gland disorders: a systematic review. European Archives of Oto-Rhino-Laryngology. 2018;275(2):347-356. doi:10.1007/s00405-017-4830-2.

7. Strychowsky JE, Sommer DD, Gupta MK, Cohen N, Nahlieli O. Sialendoscopy for the Management of Obstructive Salivary Gland Disease A Systematic Review and

Meta-Analysis. Vol 138.; 2012. http://archotol.jamanetwork.com/.

8. Atienza G, López-Cedrún JL. Management of obstructive salivary disorders by sialendoscopy: A systematic review. British Journal of Oral and Maxillofacial Surgery. 2015;53(6):507-519. doi:10.1016/j.bjoms.2015.02.024.

9. Donaldson G, de Paiva Leite S, Hardcastle T, Ahmad Z, Morton RP. The Need for Studies on Oral Corticosteroids After Sialendoscopy for Obstructive Salivary Gland Disease: Systematic Review. Annals of Otology, Rhinology and Laryngology. 2022;131(7):805-811. doi:10.1177/00034894211045262.

10. Cung TD, Lai W, Svider PF, et al. Sialendoscopy in the Management of Radioiodine Induced Sialadenitis: A Systematic Review. Annals of Otology, Rhinology and Laryngology. 2017;126(11):768-773. doi:10.1177/0003489417732795.

11. Coca KK, Gillespie MB, Beckmann NA, Zhu R, Nelson TM, Witt RL. Sialendoscopy and Sjogren’s Disease: A Systematic Review. Laryngoscope. 2021;131(7):1474-1481. doi:10.1002/lary.29233.

12. Ramakrishna J, Strychowsky J, Gupta M, Sommer DD. Sialendoscopy for the management of juvenile recurrent parotitis: A systematic review and meta-analysis. Laryngoscope. 2015;125(6):1472-1479. doi:10.1002/lary.25029.

13. Garavello W, Redaelli M, Galluzzi F, Pignataro L. Juvenile recurrent parotitis: A systematic review of treatment studies. International Journal of Pediatric Otorhinolaryngology. 2018;112:151-157. doi:10.1016/j.ijporl.2018.07.002.

14. Gundlach P, Scherer H, Hopf J, et al. [Endoscopic-controlled laser lithotripsy of salivary calculi. In vitro studies and initial clinical use]. HNO. 1990;38(7):247-250. Accessed July 7, 2022. https://europepmc.org/article/med/2394601.

15. Konigsberger R, Feyh J, Goetz A, Schilling V, Kastenbauer E. Endoscopically controlled laser lithotripsy on sialolithiasis. Laryngo- Rhino- Otologie. 1990;69(6):322-323. doi:10.1055/s-2007-998200.

16. Arzoz E, Santiago A, Esnal F, Palomero R. Endoscopic intracorporeal lithotripsy for sialolithiasis. J Oral Maxillofac Surg. 1996;54(7):847-850. doi:10.1016/S0278-2391(96)90533-9.

17. Ito H, Baba S. Pulsed dye laser lithotripsy of submandibular gland salivary calculus. J Laryngol Otol. 1996;110(10):942-946. doi:10.1017/S0022215100135418.

18. Nahlieli O, Eliav E, Hasson O, Zagury A, Baruchin AM. Pediatric sialolithiasis. Oral Surg Oral Med Oral Pathol Oral Radiol Endod. 2000;90(6):709-712. doi:10.1067/MOE.2000.109075A.

19. Marchal F, Dulguerov P, Becker M, Barki G, Disant F, Lehmann W. Specificity of parotid sialendoscopy. Laryngoscope. 2001;111(2):264-271. doi:10.1097/00005537-200102000-00015.

20. Marchal F, Dulguero P, Lehmann W, et al. Submandibular diagnostic and interventional sialendoscopy: New procedure for ductal disorders. Annals of Otology, Rhinology and Laryngology. 2002;111(1):27-35. doi:10.1177/000348940211100105.

21. Chu DW, Chow TL, Lim BH, Kwok SPY. Endoscopic management of submandibular sialolithiasis. Surg Endosc. 2003;17(6):876-879. doi:10.1007/S00464-002-8563-X.

22. Katz P. [New techniques for the treatment of salivary lithiasis: sialoendoscopy and extracorporal lithotripsy: 1773 cases]. Annales d’oto-laryngologie et de chirurgie cervico faciale : bulletin de la Societe d’oto-laryngologie des hopitaux de Paris. 2004;121(3):123-132. doi:10.1016/S0003-438X(04)95499-0.

23. Nahlieli O, Shacham R, Shlesinger M, Eliav E. Juvenile recurrent parotitis: a new method of diagnosis and treatment. Pediatrics. 2004;114(1):9-12. doi:10.1542/PEDS.114.1.9.

24. Zenk J, Koch M, Bozzato A, Iro H. Sialoscopy--initial experiences with a new endoscope. Br J Oral Maxillofac Surg. 2004;42(4):293-298. doi:10.1016/J.BJOMS.2004.03.006.

25. Ziegler CM, Steveling H, Seubert M, Mühling J. Endoscopy: a minimally invasive procedure for diagnosis and treatment of diseases of the salivary glands. Six years of practical experience. Br J Oral Maxillofac Surg. 2004;42(1):1-7. doi:10.1016/S0266-4356(03)00188-8.

26. Koch M, Zenk J, Bozzato A, Bumm K, Iro H. Sialoscopy in cases of unclear swelling of the major salivary glands. Otolaryngol Head Neck Surg. 2005;133(6):863-868. doi:10.1016/J.OTOHNS.2005.08.005.

27. McGurk M, MacBean AD, Fan KFM, Sproat C, Darwish C. Endoscopically assisted operative retrieval of parotid stones. Br J Oral Maxillofac Surg. 2006;44(2):157-160. doi:10.1016/J.BJOMS.2005.03.026.

28. Raif J, Vardi M, Nahlieli O, Gannot I. An Er:YAG laser endoscopic fiber delivery system for lithotripsy of salivary stones. Lasers Surg Med. 2006;38(6):580-587. doi:10.1002/LSM.20344.

29. Faure F, Querin S, Dulguerov P, Froehlich P, Disant F, Marchal F. Pediatric salivary gland obstructive swelling: sialendoscopic approach. Laryngoscope. 2007;117(8):1364-1367. doi:10.1097/MLG.0B013E318068657C.

30. Kim JW, Han GS, Lee SH, Lee DY, Kim YM. Sialoendoscopic treatment for radioiodine induced sialadenitis. Laryngoscope. 2007;117(1):133-136. doi:10.1097/01.MLG.0000247776.72484.62.

31. Marchal F. A combined endoscopic and external approach for extraction of large stones with preservation of parotid and submandibular glands. Laryngoscope. 2007;117(2):373-377. doi:10.1097/MLG.0B013E31802C06E9.

32. Nahlieli O, Shacham R, Zagury A, Bar T, Yoffe B. The ductal stretching technique: an endoscopic-assisted technique for removal of submandibular stones. Laryngoscope. 2007;117(6):1031-1035. doi:10.1097/MLG.0B013E31804F8163.

33. Koch M, Iro H, Zenk J. Role of sialoscopy in the treatment of Stensen’s duct strictures. Annals of Otology, Rhinology and Laryngology. 2008;117(4):271-278. doi:10.1177/000348940811700406.

34. Modayil PC, Jacob V, Manjaly G, Watson G. Intracorporeal electrokinetic lithotripsy: an advancement in minimally invasive management of parotid duct calculus. J Laryngol Otol. 2008;122(4):428-431. doi:10.1017/S0022215107008304.

35. Papadaki ME, McCain JP, Kim K, Katz RL, Kaban LB, Troulis MJ. Interventional sialoendoscopy: early clinical results. J Oral Maxillofac Surg. 2008;66(5):954-962. doi:10.1016/J.JOMS.2008.01.017.

36. Quenin S, Plouin-Gaudon I, Marchal F, Froehlich P, Disant F, Faure F. Juvenile Recurrent Parotitis: Sialendoscopic Approach. Archives of Otolaryngology-Head & Neck Surgery. 2008;134(7):715-719. doi:10.1001/ARCHOTOL.134.7.715.

37. Schmitz S, Zengel P, Alvir I, Andratschke M, Berghaus A, Lang S. Long-term evaluation of extracorporeal shock wave lithotripsy in the treatment of salivary stones. The Journal of Laryngology & Otology. 2008;122(1):65-71. doi:10.1017/S0022215107007396.

38. Walvekar RR, Razfar A, Carrau RL, Schaitkin B. Sialendoscopy and associated complications: a preliminary experience. Laryngoscope. 2008;118(5):776-779. doi:10.1097/MLG.0B013E318165E355.

39. Yu C, Zheng L, Yang C, Shen N. Causes of chronic obstructive parotitis and management by sialoendoscopy. Oral Surg Oral Med Oral Pathol Oral Radiol Endod. 2008;105(3):365-370. doi:10.1016/J.TRIPLEO.2007.08.008.

40. Yu CQ, Yang C, Zheng LY, Wu DM, Zhang J, Yun B. Selective management of obstructive submandibular sialadenitis. Br J Oral Maxillofac Surg. 2008;46(1):46-49. doi:10.1016/J.BJOMS.2007.06.008.

41. Bomeli SR, Schaitkin B, Carrau RL, Walvekar RR. Interventional sialendoscopy for treatment of radioiodine-induced sialadenitis. Laryngoscope. 2009;119(5):864-867. doi:10.1002/LARY.20140.

42. Iro H, Zenk J, Escudier MP, et al. Outcome of minimally invasive management of salivary calculi in 4,691 patients. Laryngoscope. 2009;119(2):263-268. doi:10.1002/LARY.20008.

43. Liu D gao, Zhang Z yan, Zhang Y, Zhang L, Yu G yan. Diagnosis and management of sialolithiasis with a semirigid endoscope. Oral Surg Oral Med Oral Pathol Oral Radiol Endod. 2009;108(1):9-14. doi:10.1016/J.TRIPLEO.2009.01.004.

44. Nahlieli O. Endoscopic surgery of the salivary glands. Alpha Omegan. 2009;102(2):55-60. doi:10.1016/J.AODF.2009.04.010.

45. Shacham R, Droma EB, London D, Bar T, Nahlieli O. Long-term experience with endoscopic diagnosis and treatment of juvenile recurrent parotitis. J Oral Maxillofac Surg. 2009;67(1):162-167. doi:10.1016/J.JOMS.2008.09.027.

46. Walvekar RR, Bomeli SR, Carrau RL, Schaitkin B. Combined approach technique for the management of large salivary stones. Laryngoscope. 2009;119(6):1125-1129. doi:10.1002/LARY.20203.

47. Ardekian L, Shamir D, Trabelsi M, Peled M. Chronic obstructive parotitis due to strictures of Stenson’s duct ‒ our treatment experience with sialoendoscopy. J Oral Maxillofac Surg. 2010;68(1):83-87. doi:10.1016/J.JOMS.2009.08.019.

48. Escudier MP, Brown JE, Putcha V, Capaccio P, McGurk M. Factors influencing the outcome of extracorporeal shock wave lithotripsy in the management of salivary calculi. Laryngoscope. 2010;120(8):1545-1549. doi:10.1002/LARY.21000.

49. Jabbour N, Tibesar R, Lander T, Sidman J. Sialendoscopy in children. Int J Pediatr Otorhinolaryngol. 2010;74(4):347-350. doi:10.1016/J.IJPORL.2009.12.013.

50. Karavidas K, Nahlieli O, Fritsch M, McGurk M. Minimal surgery for parotid stones: a 7-year endoscopic experience. Int J Oral Maxillofac Surg. 2010;39(1):1-4. doi:10.1016/J.IJOM.2009.06.030.

51. Koch M, Bozzato A, Iro H, Zenk J. Combined endoscopic and transcutaneous approach for parotid gland sialolithiasis: indications, technique, and results. Otolaryngol Head Neck Surg. 2010;142(1):98-103. doi:10.1016/J.OTOHNS.2009.10.022.

52. Martins-Carvalho C, Plouin-Gaudon I, Quenin S, et al. Pediatric sialendoscopy: a 5-year experience at a single institution. Arch Otolaryngol Head Neck Surg. 2010;136(1):33-36. doi:10.1001/ARCHOTO.2009.184.

453. Nahlieli O, Shacham R, Zaguri A. Combined external lithotripsy and endoscopic techniques for advanced sialolithiasis cases. J Oral Maxillofac Surg. 2010;68(2):347-353. doi:10.1016/J.JOMS.2009.09.041.

54. Serbetci E, Sengor GA. Sialendoscopy: experience with the first 60 glands in Turkey and a literature review. Ann Otol Rhinol Laryngol. 2010;119(3):155-164. doi:10.1177/000348941011900303.

55. Su Y xiong, Liao G qing, Zheng G sen, Liu H chao, Liang Y jie, Ou D ming. Sialoendoscopically assisted open sialolithectomy for removal of large submandibular hilar calculi. J Oral Maxillofac Surg. 2010;68(1):68-73. doi:10.1016/J.JOMS.2009.06.031.

56. Wallace E, Tauzin M, Hagan J, Schaitkin B, Walvekar RR. Management of giant sialoliths: review of the literature and preliminary experience with interventional sialendoscopy. Laryngoscope. 2010;120(10):1974-1978. doi:10.1002/LARY.21082.

57. Yu C, Yang C, Zheng L, Wu D. Endoscopic observation and strategic management of obstructive submandibular sialadenitis. J Oral Maxillofac Surg. 2010;68(8):1770-1775. doi:10.1016/J.JOMS.2009.09.118

58. Bowen MA, Tauzin M, Kluka EA, et al. Diagnostic and interventional sialendoscopy: a preliminary experience. Laryngoscope. 2011;121(2):299-303. doi:10.1002/LARY.21390.

59. Capaccio P, Clemente IA, McGurk M, Bossi A, Pignataro L. Transoral removal of hiloparenchymal submandibular calculi: a long-term clinical experience. Eur Arch Otorhinolaryngol. 2011;268(7):1081-1086. doi:10.1007/S00405-011-1508-Z.

60. Danquart J, Wagner N, Arndal H, Homøe P. Sialoendoscopy for diagnosis and treatment of non-neoplastic obstruction in the salivary glands. Danish Medical Bulletin. 2011;58(2):A4232-A4232. Accessed July 7, 2022. https://europepmc.org/article/med/21299921.

61. Gary C, Kluka EA, Schaitkin B, Walvekar RR. Interventional sialendoscopy for treatment of juvenile recurrent parotitis. J Indian Assoc Pediatr Surg. 2011;16(4):132-136. doi:10.4103/0971-9261.86865.

62. Gillespie MB, Intaphan J, Nguyen SA. Endoscopic-assisted management of chronic sialadenitis. Head Neck. 2011;33(9):1346-1351. doi:10.1002/HED.21620.

63. Konstantinidis I, Chatziavramidis A, Tsakiropoulou E, Malliari H, Constantinidis J. Pediatric sialendoscopy under local anesthesia: limitations and potentials. Int J Pediatr Otorhinolaryngol. 2011;75(2):245-249. doi:10.1016/J.IJPORL.2010.11.009.

64. Luers JC, Grosheva M, Stenner M, Beutner D. Sialoendoscopy: prognostic factors for endoscopic removal of salivary stones. Arch Otolaryngol Head Neck Surg. 2011;137(4):325-329. doi:10.1001/ARCHOTO.2010.238.

65. Maresh A, Kutler DI, Kacker A. Sialoendoscopy in the diagnosis and management of obstructive sialadenitis. Laryngoscope. 2011;121(3):495-500. doi:10.1002/LARY.21378.

66. Shacham R, Puterman MB, Ohana N, Nahlieli O. Endoscopic treatment of salivary glands affected by autoimmune diseases. J Oral Maxillofac Surg.2011;69(2):476-481. doi:10.1016/J.JOMS.2010.10.002.

67. Capaccio P, Sigismund PE, Luca N, Marchisio P, Pignataro L. Modern management of juvenile recurrent parotitis. J Laryngol Otol. 2012;126(12):1254-1260. doi:10.1017/S0022215112002319.

68. Durbec M, Dinkel E, Vigier S, Disant F, Marchal F, Faure F. Thulium-YAG laser sialendoscopy for parotid and submandibular sialolithiasis. Lasers Surg Med. 2012;44(10):783-786. doi:10.1002/LSM.22094.

69. Hackett AM, Baranano CF, Reed M, Duvvuri U, Smith RJ, Mehta D. Sialoendoscopy for the Treatment of Pediatric Salivary Gland Disorders. Archives of Otolaryngology-Head & Neck Surgery. 2012;138(10):912-915. doi:10.1001/2013.JAMAOTO.244.

70. Koch M, Iro H, Klintworth N, Psychogios G, Zenk J. Results of minimally invasive gland-preserving treatment in different types of parotid duct stenosis. Arch Otolaryngol Head Neck Surg. 2012;138(9):804-810. doi:10.1001/ARCHOTO.2012.1618.

71. Koch M, Iro H, Künzel J, Psychogios G, Bozzato A, Zenk J. Diagnosis and gland-preserving minimally invasive therapy for Wharton’s duct stenoses. Laryngoscope. 2012;122(3):552-558. doi:10.1002/LARY.22452.

72. Kopeć T, Szyfter W, Wierzbicka M. Sialoendoscopy and combined approach for the management of salivary gland stones. European Archives of Oto-Rhino-Laryngology. 2013;270(1):219-223. doi:10.1007/S00405-012-2145-X/FIGURES/2.

73. Prendes BL, Orloff LA, Eisele DW. Therapeutic Sialendoscopy for the Management of Radioiodine Sialadenitis. Archives of Otolaryngology-Head & Neck Surgery. 2012;138(1):15-19. doi:10.1001/ARCHOTO.2011.215.

74. Eva Rye Rasmussen, Helge Arndal, Stig Hebbelstrup Rasmussen, Niels Wagner. Steady progress seen in endoscopic surgery on major salivary glands. Danish Medical Journal. 2012;59(11).

75. Zenk J, Koch M, Klintworth N, et al. Sialendoscopy in the diagnosis and treatment of sialolithiasis: A study on more than 1000 patients. Otolaryngology-Head and Neck Surgery (United States). 2012;147(5):858-863. doi:10.1177/0194599812452837.

76. Carroll WW, Walvekar RR, Gillespie MB. Transfacial ultrasound-guided gland-preserving resection of parotid sialoliths. Otolaryngol Head Neck Surg. 2013;148(2):229-234. doi:10.1177/0194599812471514.

77. Koch M, Iro H, Zenk J. Combined endoscopic-transcutaneous surgery in parotid gland sialolithiasis and other ductal diseases: reporting medium- to long-term objective and patients’ subjective outcomes. Eur Arch Otorhinolaryngol. 2013;270(6):1933-1940. doi:10.1007/S00405-012-2286-Y.

78. Kopeć T, Wierzbicka M, Szyfter W, Leszczyńska M. Algorithm changes in treatment of submandibular gland sialolithiasis. Eur Arch Otorhinolaryngol. 2013;270(7):2089-2093. doi:10.1007/S00405-013-2463-7.

79. Kopeć T, Szyfter W, Wierzbicka M, Nealis J. Stenoses of the salivary ducts-sialendoscopy based diagnosis and treatment. Br J Oral Maxillofac Surg. 2013;51(7). doi:10.1016/J.BJOMS.2012.08.004.

80. Kopeć T, Szyfter W, Wierzbicka M. Sialoendoscopy and combined approach for the management of salivary gland stones. Eur Arch Otorhinolaryngol. 2013;270(1):219-223. doi:10.1007/S00405-012-2145-X.

81. Kroll T, Finkensieper M, Sharma SJ, Guntinas-Lichius O, Wittekindt C. Short-term outcome and patient satisfaction after sialendoscopy. Eur Arch Otorhinolaryngol. 2013;270(11):2939-2945. doi:10.1007/S00405-013-2418-Z.

82. Martellucci S, Pagliuca G, de Vincentiis M, et al. Ho:Yag laser for sialolithiasis of Wharton’s duct. Otolaryngol Head Neck Surg. 2013;148(5):770-774. doi:10.1177/0194599813479914.

83. Meyer A, Delas B, Hibon R, Faure F, Dehesdin D, Choussy O. Sialendoscopy: a new diagnostic and therapeutic tool. Eur Ann Otorhinolaryngol Head Neck Dis. 2013;130(2):61-65. doi:10.1016/J.ANORL.2012.02.010.

84. Schneider H, Koch M, Künzel J, et al. Juvenile recurrent parotitis: A retrospective comparison of sialendoscopy versus conservative therapy. Laryngoscope. 2014;124(2):451-455. doi:10.1002/LARY.24291.

85. Vashishta R, Gillespie MB. Salivary endoscopy for idiopathic chronic sialadenitis. Laryngoscope. 2013;123(12):3016-3020. doi:10.1002/LARY.24211.

86. Ardekian L, Klein HH, Araydy S, Marchal F. The use of sialendoscopy for the treatment of multiple salivary gland stones. J Oral Maxillofac Surg. 2014;72(1):89-95. doi:10.1016/J.JOMS.2013.06.206.

87. Ardekian L, Klein H, al Abri R, Marchal F. Sialendoscopy for the diagnosis and treatment of juvenile recurrent parotitis. Revue de Stomatologie, de Chirurgie Maxillo-faciale et de Chirurgie Orale. 2014;115(1):17-21. doi:10.1016/J.REVSTO.2013.12.005.

88. Pasquale C, Michele G, Lorenzo P. Sialendoscopy-assisted transfacial surgical removal of parotid stones. J Craniomaxillofac Surg. 2014;42(8):1964-1969. doi:10.1016/J.JCMS.2014.08.009.

89. de Luca R, Vicidomini A, Trodella M, Tartaro G, Colella G. Sialoendoscopy: a viable treatment for I(131) induced sialoadenitis. Br J Oral Maxillofac Surg. 2014;52(7):641-646. doi:10.1016/J.BJOMS.2014.01.025.

90. Desmots F, Chossegros C, Salles F, Gallucci A, Moulin G, Varoquaux A. Lithotripsy for salivary stones with prospective US assessment on our first 25 consecutive patients. Journal of Cranio-Maxillofacial Surgery. 2014;42(5):577-582. doi:10.1016/J.JCMS.2013.07.029.

91. Ianovski I, Morton RP, Ahmad Z. Patient-perceived outcome after sialendoscopy using the glasgow benefit inventory. Laryngoscope. 2014;124(4):869-874. doi:10.1002/LARY.24343.

92. Klein H, Ardekian L. The treatment of large sialoliths by sialendoscopic combined approach. J Oral Maxillofac Surg. 2014;72(4):737-743. doi:10.1016/J.JOMS.2013.09.003.

93. Konstantinidis I, Chatziavramidis A, Iakovou I, Constantinidis J. Long-term results of combined approach in parotid sialolithiasis. Eur Arch Otorhinolaryngol. 2015;272(11):3533-3538. doi:10.1007/S00405-014-3391-X.

94. Mikolajczak S, Meyer MF, Beutner D, Luers JC. Treatment of chronic recurrent juvenile parotitis using sialendoscopy. Acta Otolaryngol. 2014;134(5):531-535. doi:10.3109/00016489.2013.879738.

95. Numminen J, Sillanpää S, Virtanen J, Sipilä M, Rautiainen M. Retrospective analysis of a combined endoscopic and transcutaneous technique for the management of parotid salivary gland stones. ORL J Otorhinolaryngol Relat Spec. 2014;76(5):282-287. doi:10.1159/000368719.

96. Phillips J, Withrow K. Outcomes of Holmium Laser-Assisted Lithotripsy with Sialendoscopy in Treatment of Sialolithiasis. Otolaryngol Head Neck Surg. 2014;150(6):962-967. doi:10.1177/0194599814524716.

97. Semensohn R, Spektor Z, Kay DJ, Archilla AS, Mandell DL. Pediatric sialendoscopy: initial experience in a pediatric otolaryngology group practice. Laryngoscope. 2015;125(2):480-484. doi:10.1002/LARY.24868.

98. Sionis S, Caria RA, Trucas M, Brennan PA, Puxeddu R. Sialoendoscopy with and without holmium:YAG laser-assisted lithotripsy in the management of obstructive sialadenitis of major salivary glands. Br J Oral Maxillofac Surg. 2014;52(1):58-62. doi:10.1016/J.BJOMS.2013.06.015.

99. Bhayani MK, Acharya V, Kongkiatkamon S, et al. Sialendoscopy for Patients with Radioiodine-Induced Sialadenitis and Xerostomia. Thyroid. 2015;25(7):834-838. doi:10.1089/THY.2014.0572.

100 de Luca R, Trodella M, Vicidomini A, Colella G, Tartaro G. Endoscopic management of salivary gland obstructive diseases in patients with Sjögren’s syndrome. J Craniomaxillofac Surg. 2015;43(8):1643-1649. doi:10.1016/J.JCMS.2015.06.036.

101. Mikolajczak S, Bremke M, Beutner D, Luers JC. Combined endoscopic and transcutaneous approach for immobile parotid stones. Acta Otolaryngol. 2015;135(1):85-89. doi:10.3109/00016489.2014.953204.

102. Nahlieli O. Complications of Sialendoscopy: Personal Experience, Literature Analysis, and Suggestions. Journal of Oral and Maxillofacial Surgery. 2015;73(1):75-80. doi:10.1016/J.JOMS.2014.07.028.

103. Papadopoulou-Alataki E, Chatziavramidis A, Vampertzi O, Alataki S, Konstantinidis I. Evaluation and management of juvenile recurrent parotitis in children from northern Greece. Hippokratia. 2015;19(4):356. Accessed July 7, 2022. /pmc/articles/PMC5033148/.

104. Rosbe KW, Milev D, Chang JL. Effectiveness and costs of sialendoscopy in pediatric patients with salivary gland disorders. Laryngoscope. 2015;125(12):2805-2809. doi:10.1002/LARY.25384.

105. C H Su, K S Lee, T M Tseng, S H Hung. Endoscopic Holmium:YAG laser-assisted lithotripsy: A Preliminary Report. B-ENT. Published online 2015:57-61. Accessed July 7, 2022. http://www.b-ent.be/Content/files/sayilar/26/2015-11-1-057.pdf.

106. Wu C bin, Xi H, Zhou Q, Zhang LM. Sialendoscopy-assisted treatment for radioiodine-induced sialadenitis. J Oral Maxillofac Surg. 2015;73(3):475-481. doi:10.1016/J.JOMS.2014.09.025.

107. Cordesmeyer R, Winterhoff J, Kauffmann P, Laskawi R. Sialoendoscopy as a diagnostic and therapeutic option for obstructive diseases of the large salivary glands ‒ a retrospective analysis. Clinical Oral Investigations. 2016;20(5):1065-1070. doi:10.1007/S00784-015-1588-Z/TABLES/3.

108. Honnet S, Edkins O. Sialendoscopic treatment of recurrent juvenile parotitis: A South African case series. S Afr Med J. 2016;106(8):809-812. doi:10.7196/SAMJ.2016.V106I8.10561.

109. Jager DJ, Karagozoglu KH, Maarse F, Brand HS, Forouzanfar T. Sialendoscopy of Salivary Glands Affected by Sjögren Syndrome: A Randomized Controlled Pilot Study. J Oral Maxillofac Surg. 2016;74(6):1167-1174. doi:10.1016/J.JOMS.2015.12.019.

110. Kim YM, Choi JS, Hong S bin, Hyun IY, Lim JY. Salivary gland function after sialendoscopy for treatment of chronic radioiodine-induced sialadenitis. Head Neck. 2016;38(1):51-58. doi:10.1002/HED.23844.

111. Koch M, Mantsopoulos K, Schapher M, von Scotti F, Iro H. Intraductal pneumatic lithotripsy for salivary stones with the StoneBreaker: Preliminary experience. Laryngoscope. 2016;126(7):1545-1550. doi:10.1002/LARY.25849.

112. C-H Su, H Tseng, K-S Lee, T-M Tseng, S-H Hung. Experiences in the treatment of obstructive sialoadenitis with sialendoscopy. B-ENT. Published online 2016:199-206.

113. Achim V, Light TJ, Andersen PE. Gland Preservation in Patients Undergoing Sialoendoscopy. *Otolaryngol Head Neck Surg*. 2017;157(1):53-57. doi:10.1177/0194599817695547.

114. Capaccio P, Canzi P, Gaffuri M, et al. Modern management of paediatric obstructive salivary disorders: long-term clinical experience La gestione moderna dei disordini ostruttivi salivari in età pediatrica: esperienza clinica a lungo termine. *ACTA OTORHINOLARYNGOLOGICA ITALICA*. 2017;37:160-167. doi:10.14639/0392-100X-1607.

115. Carta F, Farneti P, Cantore S, et al. Sialendoscopy for salivary stones: principles, technical skills and therapeutic experience. Acta Otorhinolaryngologica Italica. 2017;37(2):102. doi:10.14639/0392-100X-1599.

116. Guo YF, Sun NN, Wu C bin, Xue L, Zhou Q. Sialendoscopy-assisted treatment for chronic obstructive parotitis related to Sjogren syndrome. Oral Surg Oral Med Oral Pathol Oral Radiol. 2017;123(3):305-309. doi:10.1016/J.OOOO.2016.10.011.

117. Singh PP, Goyal M, Goyal A. Sialendoscopic Approach in Management of Juvenile Recurrent Parotitis. Indian Journal of Otolaryngology and Head and Neck Surgery. 2017;69(4):453-458. doi:10.1007/S12070-017-1223-1/TABLES/2.

118. Bawazeer N, Carvalho J, Djennaoui I, Charpiot A. Sialendoscopy under conscious sedation versus general anesthesia. A comparative study. Am J Otolaryngol. 2018;39(6):754-758. doi:10.1016/J.AMJOTO.2018.09.002.

119. Berlucchi M, Rampinelli V, Ferrari M, Grazioli P, Redaelli De Zinis LO. Sialoendoscopy for treatment of juvenile recurrent parotitis: The Brescia experience. International Journal of Pediatric Otorhinolaryngology. 2018;105:163-166. doi:10.1016/J.IJPORL.2017.12.024

120. Capaccio P, Canzi P, Torretta S, et al. Combined interventional sialendoscopy and intraductal steroid therapy for recurrent sialadenitis in Sjögren’s syndrome: Results of a pilot monocentric trial. Clin Otolaryngol. 2018;43(1):96-102. doi:10.1111/COA.12911.

121. Hakki Karagozoglu K, Vissink A, Forouzanfar T, Brand HS, Maarse F, Jan Jager DH. Sialendoscopy enhances salivary gland function in Sjögren’s syndrome: a 6-month follow-up, randomised and controlled, single blind study. Ann Rheum Dis. 2018;77(7):1025-1031. doi:10.1136/ANNRHEUMDIS-2017-212672.

122. Guenzel T, Hoch S, Heinze N, et al. Sialendoscopy plus laser lithotripsy in sialolithiasis of the submandibular gland in 64 patients: A simple and safe procedure. Auris Nasus Larynx. 2019;46:797-802.

123. Özçelik N, Altın G, Medipol Üniversitesi Tıp Fakültesi Kulak Burun Boğaz Anabilim Dalı İ. Holmium:yttrium aluminum garnet laser lithotripsy for salivary calculi: A preliminary experience of 31 procedures. 2019;29(1):47-51.

124. de Paiva Leite SH, Morton RP, Ahmad Z, Marchal F. Do Postoperative Oral Corticosteroids Improve Results After Sialendoscopy for Ductal Stenosis? Laryngoscope. 2021;131:E1503-E1509.
